# Functional Characterization of d*rim2*, the *Drosophila melanogaster* Homolog of the Yeast Mitochondrial Deoxynucleotide Transporter*[Fn FN2]

**DOI:** 10.1074/jbc.M113.543926

**Published:** 2014-01-27

**Authors:** Caterina Da-Rè, Elisa Franzolin, Alberto Biscontin, Antonia Piazzesi, Beniamina Pacchioni, Maria Cristina Gagliani, Gabriella Mazzotta, Carlo Tacchetti, Mauro A. Zordan, Massimo Zeviani, Paolo Bernardi, Vera Bianchi, Cristiano De Pittà, Rodolfo Costa

**Affiliations:** From the ‡ Department of Biology, and; § CRIBI Biotechnology Centre, University of Padova, 35131 Padova, Italy,; the ¶ MicroScoBio Research Center, Department of Experimental Medicine, University of Genova, 16132 Genova, Italy,; the ** MRC Mitocondrial Biology Unit, University of Cambridge, Cambridge CB2 0XY, United Kingdom,; the ‡‡ Department of Biomedical Sciences, University of Padova, 35131 Padova, Italy, and; the ‖ Experimental Imaging Center, San Raffaele Scientific Institute, 20132 Milano, Italy

**Keywords:** Drosophila, Mitochondria, Mitochondrial DNA, Mitochondrial Transport, Nucleotide

## Abstract

The *CG18317* gene (d*rim2*) is the *Drosophila melanogaster* homolog of the *Saccharomyces cerevisiae Rim2* gene, which encodes a pyrimidine (deoxy)nucleotide carrier. Here, we tested if the d*rim2* gene also encodes for a deoxynucleotide transporter in the fruit fly. The protein was localized to mitochondria. *Drosophila* S2R^+^ cells, silenced for d*rim2* expression, contained markedly reduced pools of both purine and pyrimidine dNTPs in mitochondria, whereas cytosolic pools were unaffected. *In vivo* d*rim2* homozygous knock-out was lethal at the larval stage, preceded by the following: (i) impaired locomotor behavior; (ii) decreased rates of oxygen consumption, and (iii) depletion of mtDNA. We conclude that the *Drosophila* mitochondrial carrier dRIM2 transports all DNA precursors and is essential to maintain mitochondrial function.

## Teaser

**Background:** Carrier-mediated influx of cytosolic deoxynucleotides is a major source of precursors for mitochondrial DNA synthesis.

**Results:** dRIM2 is required to maintain normal deoxynucleotide pools in *Drosophila* mitochondria, and its knock-out is lethal at the larval stage.

**Conclusion:** dRIM2 is a deoxynucleotide carrier and is essential to maintain mitochondrial function.

**Significance:** Our data provide the first animal model of RIM2 deficiency.

## Introduction

Deoxy- and ribonucleoside triphosphates (dNTPs and rNTPs) are essential for the replication and transcription of the mitochondrial genome. An appropriate supply of these precursors is thus necessary for the maintenance of functional mitochondria throughout the life of cells and organisms (1, 2). The main site of deoxy- and ribonucleotide synthesis is the cytoplasm where they are produced by two *de novo* pathways interconnected through the ribonucleoside diphosphates, which are both the immediate precursors of rNTPs and the substrates for ribonucleotide reductase, the key enzyme in the *de novo* synthesis of dNTPs (3). *De novo* synthesis of thymidylate, the first committed step of thymidine deoxynucleotide *de novo* synthesis, occurs in the nucleus and in the mitochondrial matrix (4, 5). In most but not all organisms, NTPs and dNTPs are also synthesized by salvage of (deoxy)nucleosides by dedicated nucleoside and nucleotide kinases. Mammals contain two parallel deoxynucleoside salvage pathways, located in the cytosol and in mitochondria, respectively. The rate-limiting enzymes are two cytosolic and two mitochondrial (mt)^3^ deoxynucleoside kinases whose combined substrate specificities permit the salvage of all deoxynucleosides in each of the two subcellular compartments (6). Ribonucleotide salvage consists primarily in the recycling of ribonucleosides and free purine bases and occurs in the cytoplasm.

The nuclear envelope is freely permeable to nucleotides, and the precursors made in the cytoplasm are therefore easily available for nuclear DNA replication and transcription. The mt inner membrane is instead impermeable to nucleotides, and cytosolic nucleotides need membrane carriers to reach the mt matrix where mtDNA transactions take place. At present, only a few mt nucleotide carriers are known (7). In yeast, they include the three isoforms of the ATP/ADP exchanger, a GTP/GDP carrier (Ggc1p) (8) and a (deoxy)nucleotide carrier (Rim2p) (9). Two human genes, *SLC25A33* and *SLC25A36* (7, 10), were suggested to code for nucleotide carriers on the basis of their homology to the *Saccharomyces cerevisiae RIM2*. The prediction has been confirmed only in the case of *SLC25A33* (11, 12).

The product of *SLC25A33* is a protein 33% identical to Rim2p. It has been named PNC1 (11) to highlight its function as a pyrimidine nucleotide carrier, demonstrated by transport studies with the recombinant protein reconstituted in liposomes (11) and later by isotope flow experiments in intact cells (12). The transport properties of yeast Rim2p and human PNC1 are very similar. When reconstituted into liposomes, both proteins exchange all pyrimidine ribo- and deoxyribonucleotides and show some activity with guanine nucleotides but not with adenine nucleotides. Thus, they appear to be responsible for the import of most nucleic acid precursors into mitochondria. The characterization of Rim2p activity led to the proposal that the carrier imports nucleoside triphosphates in exchange with monophosphates (9).

Rim2p deletion had been found to cause total loss of mtDNA in yeast long before the transport activity of the protein had been identified biochemically (13). So far, there are no data on the *in vivo* effects of PNC1 loss of function in humans or mice. The existing information comes from experiments of PNC1 silencing by siRNA in cultured human cells. Knockdown (KD) of PNC1 led to depletion of mtDNA and reduced transcription of mt genes and impairment of oxidative phosphorylation (11, 14). *In situ* analysis of nucleotide flow in cells with down-regulation of PNC1 revealed a slower mitochondrial uptake of uridine triphosphate and a slower release of thymidine nucleotides to the cytoplasm (12). The same study investigated also the function of the *SLC25A36* gene product. Down-regulation of the protein, which is 60% identical to PNC1, had no effect on mitochondrial pools. Therefore, the activity of SLC25A36 remains unknown (12).

The genome of *Drosophila melanogaster* contains only one gene with significant similarity to yeast *Rim2* and the two human genes. This gene, denominated *CG183173* and indicated here as d*rim2,* maps on chromosome 2 (position 22B1), spans 7,503 bp, and produces three different transcripts, all containing the typical features of mitochondrial carriers (15). There is currently no information concerning the functions of d*rim2*. On account of its homology to Rim2p and PNC1, it may be involved in the mitochondrial transport of nucleotides. We considered that it could be a useful model to investigate how the deletion of a nucleotide carrier affects mitochondrial function in a multicellular animal.

We first silenced d*rim2* expression in the *Drosophila* S2R^+^ cell line (16) and found depletion of all mitochondrial dNTP pools, suggesting that the protein is involved in the transport of all four DNA precursors. We then produced d*rim2* knock-out (KO) flies and found that the homozygous loss of d*rim2* is lethal, blocking larval development at the third instar. We analyzed different phenotypic aspects of the d*rim2*^−/−^ larvae detecting profound alterations of mitochondrial structure and function and impairment of larval locomotion that could be related to depletion of mtDNA. Our data suggest that d*rim2* codes for a nucleotide transporter essential for the maintenance of functional mitochondria in *Drosophila*.

## EXPERIMENTAL PROCEDURES

### 

#### 

##### Cell Cultures

The *Drosophila* S2R^+^ cell line was derived from a primary culture of late stage (20–24 h old) *D. melanogaster* embryos (16). It was obtained from *Drosophila* Genomics Resource Center. S2R^+^ cells grow at 25 °C without CO_2_ in Schneider's medium (Invitrogen) with 10% heat-inactivated fetal bovine serum (FBS) (Sigma) as a loose semi-adherent monolayer, showing a doubling time of about 48 h.

##### dsRNA Production and RNAi Procedures

dsRNAi synthesis was performed employing the T7 Megascript kit (Invitrogen) (17, 18). The oligonucleotide primers used to synthesize dsRNA starting from cDNA were d*rim2_*T7 forward (F) and reverse (R) (primer sequences are reported in Table 1). These primers give two complementary 700-bp RNA products that anneal as temperature decreases, forming a final 700-bp dsRNA. About 2 × 10^6^ cells suspended in 1 ml of serum-free medium were mixed with 2 μg/ml dsRNA, plated in a 24-well plate, and incubated at room temperature (RT) for 1 h. Subsequently, 1 volume of complete medium (2×) was added, and cells were grown in the presence of dsRNA for 2 days at 25 °C.

**TABLE 1:** **Sequences of oligonucleotides used in this study** Primer sequences are indicated as 5′**–**3′ direction.

Primer name	Sequence 5′–3′
d*rim2_*T7 F	taatacgactcactatagggagatgtatgcgttttgccaaagttaaa
d*rim2_*T7 R	taatacgactcactatagggagaatggtacaccctctcgatgcactg
d*rim2*KO^e01575^F	atttcgcctcacagctttg
d*rim2*KO^e01575^R	gatgacaaagtgcaccatcg
d*rim2*KO^e00041^F	ctcttcgattctgggcattc
d*rim2*KO^e00041^R	tactattcctttcactcgcacttattg
d*rim2* F	tcggttacggatcgaacaa
d*rim2*R	tcggttacggatcgaacaa
*CoxI* F	tgctcctgatatagcattcccacga
*CoxI* R	tccaccatgagcaattccagcgg
*16S* F	aaaaagattgcgacctcgat
*16S* R	aaaccaacctggcttacacc
*Rp49* F	tcggttacggatcgaacaa
*Rp49* R	gacaatctccttgcgcttct
*Rpl32* F	aggcccaagatcgtgaagaa
*Rpl32* R	tgtgcaccaggaacttcttgaa

##### dNTP Pool Extraction and Analysis

At the end of the treatment with dsRNA, about 10 × 10^6^ S2R^+^ cells were centrifuged in 15-ml tubes for 10 min at 400 × *g*, and the pellet was washed twice with ice-cold PBS. The cells were then resuspended in 200 μl of extraction buffer (0.21 m mannitol, 0.07 m sucrose, 0.2 m EGTA, 10 mm Tris-HCl, pH 7.5, 0.5% BSA), and a suspension of glass beads (0.1 mm diameter) corresponding to about ½ the volume of the cellular pellet was added. The cell/bead suspension was introduced in a Bullet Blender Storm homogenizer (Next Advance) and shaken for 2 min at speed 8, and then 400 μl of extraction buffer were added, and the glass beads were removed by a short centrifugation. Mitochondrial and cytosolic nucleotide pools were isolated from the whole cell homogenate by differential centrifugation and methanol extraction as described (19). All manipulations took place in a cold room. The pellet remaining after mitochondrial pool extraction was dissolved in 1 ml of 0.3 m NaOH. The *A*_260 nm_ of the NaOH fraction was used to normalize the number of cells from which the pools of the different samples were extracted (20). The sizes of the dNTP pools were determined with a DNA polymerase-based assay (21) with the modifications reported previously (22). Two different aliquots of each pool extract were analyzed, and pool sizes were expressed as pmol of dNTPs/million cells, with cell numbers calculated as indicated above.

##### Immunolocalization of dRIM2

HA-tagged d*rim2* cDNA was cloned in a pACT vector under the control of *actin 5c* promoter (23). 500,000 cells were seeded on round coverslips and grown for 16 h, and then they were transfected with 20 μl of CellFectin II reagent and 2.5 μg of vector and incubated for 8 h. The medium was removed and replaced with complete Schneider medium (containing 10% heat-inactivated FBS). 48 h from transfection, cells were washed once with 1× PBS and incubated with 100 nm MitoTracker Red CMXROS and 1 μg/ml cyclosporin H in Schneider's Medium for 20 min (23). Then the cells were washed in 1× PBS and fixed in 4% paraformaldehyde for 20 min. After a second wash in PBS, cells were permeabilized for 5 min with 50 mm NH_4_Cl in PBS + 0.1% Triton X-100, then blocked for 1 h in 3% goat serum in PBS, washed again, and incubated with 1:100 monoclonal mouse α-HA antibody (Sigma) at 4 °C overnight. Cells were washed again with PBS and then incubated with 1:500 FITC-conjugated α-mouse IgG (Sigma) with 2% goat serum for 45 min. After a final wash in PBS, the slides were mounted with Vectashield mounting medium. Images were taken with a Leica SP5 confocal microscope at ×63 magnification.

##### Fly Stocks and Breeding Conditions

Flies were raised on standard cornmeal medium and were maintained at 23 °C, 70% relative humidity, on a 12-h light/dark cycle. The UAS fly strains (transformant IDs 44203 and 44202) used to perform post-transcriptional silencing were from the Vienna *Drosophila* RNAi Center. Other *D. melanogaster* strains were obtained from the Bloomington Stock Center.

##### Egg-to-Adult Viability

For each of the transgenic lines, around 300 fertilized eggs were collected on standard yeast/glucose/agar medium in a Petri dish (60 × 15 mm). The fertilized eggs were incubated at 23 °C, and for each experimental condition, the number of individuals reaching the third instar larva, pupa, or adult, and the relative percentages were calculated (24).

##### Genomic DNA Extraction

Single individuals (flies or larvae) were homogenized in separate vials in 50 μl of extraction buffer containing 10 mm Tris-HCl, pH 8.2, 1 mm EDTA, and 25 mm NaCl. Proteinase K was added to a final concentration of 200 μg/ml, and the homogenate was incubated for 45 min at 37 °C followed by heat inactivation of the enzyme at 95 °C for 5 min. Genomic DNA used to quantify the mtDNA copy number was extracted from 10 larvae using the phenol/chloroform DNA extraction protocol.

##### Primers

All oligonucleotides used in this work are reported in the Table 1. They were designed using the on-line tool Primer-BLAST (25).

##### Generation of Knock-out Strain

We obtained from Exelixis *Drosophila* Stock Center available stocks bearing PiggyBac insertions (PBac(RB) CG18317^e01575^ and PBac(RB) CG18317^e00041^) at the boundaries of the d*rim2* locus. To obtain the gene deletion, we exploited the specific recombination between the FRT element within the PBac elements catalyzed by FLP recombinase (26, 27). The obtained KO lines were checked for the presence of the deletion. To this purpose, the following couples of primers were used upstream deletion (d*rim2*KO^e01575^ F and R), giving a product size of 150 nucleotides, and downstream deletion (d*rim2*KO^e00041^ F and R), giving a product size of 240 nucleotides.

##### RNA Isolation and qRT-PCR Experiments

Total RNA was extracted from ∼10 larvae or 2 × 10^6^ cells using TRIzol (Invitrogen) and further purified by precipitation with 8 m LiCl. RNA samples were checked for integrity by capillary electrophoresis (RNA 6000Nano LabChip, Agilent Technologies). For each sample, 1 μg of RNA was used for first strand cDNA synthesis, employing 10 mm deoxynucleotides, 10 μm oligo(dT), and SuperScript II (Invitrogen). qRT-PCRs were performed in triplicate in a 7500 Real Time PCR system (Invitrogen) using SYBR Green chemistry (Promega). The 2^−ΔΔ^*^Ct^* (relative quantification) method implemented in the 7500 Real Time PCR system software was used to calculate the relative expression ratio (28). The d*rim2* oligonucleotide primer used was d*rim2* F and R. Specific primers were designed for *CoxI* (*CoxI* F and R).The *16S* primers used were *16S* F and R (29). *Rp49* was used as endogenous control, and the oligonucleotides employed were *Rp49* F and R.

##### Body Wall Preparations

A small portion of the tip was cut from third instar larvae; internal organs were removed by gently squeezing from end to end, and the preparation was turned inside-out by rolling the cuticula along a holding tweezer.

##### Mitochondrial Pattern in Muscle Fibers

Body wall preparations of larvae were stained with 500 nm MitoTracker Red CMXRos (Invitrogen) for 45 min. Tissues were then washed in 1× PBS and fixed in 4% paraformaldehyde for 20 min. After a brief wash, they were mounted in 80% glycerol. Scans of muscle six were taken with a Leica SP5 Confocal Microscope at ×63 magnification.

##### Measurements of Oxygen Consumption

Oxygen measurements were made using the XF24 Extracellular flux analyzer (Seahorse Bioscience). Measurements were performed both in whole tissues of *Drosophila* larvae (body wall preparations) and in S2R^+^ *Drosophila* cells, using different Seahorse technologies. The instrument was maintained at a temperature of 25 °C. In the case of tissues, each dissected larvae was placed into a well of an islet capture 24-well microplate. Islet capture screens were used to keep the larvae in place. Basal oxygen consumption rates, reported in the unit of picomoles/min, were measured several times before injecting the first drug to be tested. Chemicals were sequentially added in each well as described in figure legends. Cells were seeded onto XF-24-well plates at 20,000 cells/well and cultured for 48 h. The following day, the culture medium was replaced with serum-free Schneider medium (Invitrogen). Basal oxygen consumption rates were measured three times, and the loaded compounds were then sequentially injected.

##### Analysis of Larval Locomotor Behavior

The locomotor activity of a single third stage larva inside an arena was recorded for a 120-s period using a video tracking system. The arena consisted in a Petri dish (5 cm in diameter) covered by a thin layer of 1% agar gel. The Petri dish was placed inside a box; the internal walls of the box were painted black, containing a ring of ultrabright white leads to generate a uniform illumination. After closing the box, the movement of the larva inside the arena was video recorded using a Canon digital video camera (10 frames/s). A specific software (AnyMaze) was utilized to track the path covered by the moving animal during the recording time period of 120 s. The software calculated the total length of the path, the average speed, and the maximum speed of the larvae. A total number of 50 larvae were analyzed for each genotype. The tests were performed at the same time of day for all strains.

##### Electron Microscopy

Third stage larvae were dissected in Ca^2+^-free hemolymph**-**like saline**-**3 (HL3) and transferred into a fixation solution containing 3% glutaraldehyde, 2% paraformaldehyde, 100 mm sucrose, and 2 mm EGTA in 0.1 mm sodium phosphate buffer, pH 7.2. Samples were fixed for 2 h and washed overnight at 4 °C in 0.1 mm phosphate buffer, pH 7.2. Samples were then post-fixed for 2 h with cold 1% OsO_4_, in 0.1 mm sodium cacodylate buffer, pH 7.2, and rinsed 3–4 times (5 min each) in 0.1 mm sodium phosphate buffer, pH 7.2. Subsequently larvae were dehydrated through an ethanol gradient, followed by a propylene oxide-resin gradient. Finally, the samples were embedded in Epon resin (Sigma) and polymerized at 60 °C for 3 days. The analysis was performed on 60-nm ultrathin sections of larval body wall muscles, stained for 20 min with uranyl acetate, and examined with a Philips CM10 electron microscope (FEI Co.). For statistical analyses, the shortest and the longest diameters of mitochondria were measured using ImageJ software (rsb.info.nih.gov). In addition, we measured the area and the density of mitochondria in the same larvae. In particular, we first measured the area occupied by all cell profiles present on each section for each genotype considered. Over this area, we then measured the total number and the area occupied by mitochondria. The data were collected considering the total area occupied by cell profiles on sections cut at two different levels (about 50 μm apart) of the same block and placed on different grid. The analysis were performed using “Netherlennder” system (30).

##### Determination of mtDNA Levels

Total DNA from larvae was extracted using phenol/chloroform precipitation. The amount of mtDNA was assessed by the ratio of mtDNA to nuclear DNA (nDNA) copy number determined by quantitative real time amplification of the mitochondrial *16S* gene and the nuclear *Rpl32* gene. Primers used in this work (*16S* F and R; *Rpl32* F and R) were those reported previously (29). We generated two gene-specific calibration curves with six 10-fold serial dilutions (100–10,000,000 copies) of plasmids containing the cloned target sequences (Invitrogen). Concentration of plasmid stock solutions was assessed with an ND-1000 spectrophotometer (NanoDrop), and the plasmid copy number of dilutions was calculated using Avogadro's number. Reactions were performed in triplicate using SYBR Green chemistry according to the manufacturer's recommendations (GoTaq qPCR Master Mix, Promega) in a 7500 Real Time PCR System instrument (Invitrogen). Data were normalized to the ratio of mtDNA/nDNA copy number in controls (arbitrary set to 100%) (31, 32).

##### DNA Microarray Design

Probes were designed using the Agilent eArray Custom Microarray Design Service, which applies proprietary prediction algorithms to design 60-mer oligonucleotide probes. Microarrays were synthesized *in situ* using the Agilent ink-jet technology with 8 × 60 K format. A total of 32,162 probes representing *D. melanogaster* transcripts were successfully obtained. A custom microarray platform, named “*Drosophila* 1.0” (eArray Design ID: 035757), showed 30,814 duplicate probes and 1,348 single probes. Each array included default positive (1,011 probes) and negative (308 probes) controls. Probe sequences and other details on the microarray platform can be found in the Gene Expression Omnibus (GEO) database (www.ncbi.nlm.nih.gov) under accession number GPL17290.

##### Microarray Labeling and Hybridization

Gene expression profiling was carried out on d*rim2*^−/−^ and d*rim2*^+/−^ *Drosophila* larvae using the *Drosophila* 1.0 custom platform (Agilent Technologies). Total RNA was obtained from the whole body of third instar larvae for each genotype. Four and three biological replicates were analyzed for d*rim2*^−/−^ and d*rim2*^+/−^ samples, respectively, for a total of seven microarray experiments. 800 ng of total RNA was labeled with “Agilent One-color Microarray-based Gene Expression” protocol according to the manufacturer's instructions. The synthesized cDNA was transcribed into cRNA and labeled with Cy3-dCTP. Labeled cRNA was purified with RNeasy mini columns (Qiagen). The quality of each cRNA sample was verified by total yield, and specificity was calculated with NanoDrop ND-1000 spectrophotometer measurements. 1.65 μg of labeled cRNA was used in each reaction, and hybridization was carried out at 65 °C for 17 h in a hybridization oven rotator (Agilent). The arrays were washed using Agilent Gene expression washing buffers and stabilization and drying solution, as suggested by the supplier. Slides were scanned on an Agilent microarray scanner (model G2565CA), and Agilent Feature Extraction software version 10.5.1.1 was used for image analysis. Gene expression data are available in the GEO database with the accession number GSE48012.

##### Statistical Analysis of Gene Expression Data

Inter-array normalization of expression levels was performed with the quantile method (33) to correct possible experimental distortions. A normalization function was applied to the expression data of all the experiments, and the values of within-array replicate spots were then averaged. Feature Extraction software, which provided spot quality measures, was used to evaluate the quality and reliability of the hybridization. In particular, the flag “glsFound” (set to 1 if the spot had an intensity value significantly different from the local background and to 0 when otherwise) was used to filter out unreliable probes; the flag equal to 0 was to be noted as “not available.” Probes with a high proportion of not available values were removed from the dataset to carry out a more solid, unbiased statistical analysis. Forty percent of the not available was used as the threshold in the filtering process, and a total of 25,350 *Drosophila* transcripts were obtained. Principal component analysis, cluster analysis, and profile similarity searches were performed with MultiExperiment Viewer version 4.8.1 of the TM4 microarray software suite. The identification of differentially expressed mRNAs was performed with two class Significance Analysis of Microarray programs with default settings (34). The normalized expression values of the biological replicates for each genotype were log2-transformed and mediated. Gene Ontology analysis of differentially expressed genes was performed using the DAVID tool (35).

## RESULTS

**FIGURE 1.**
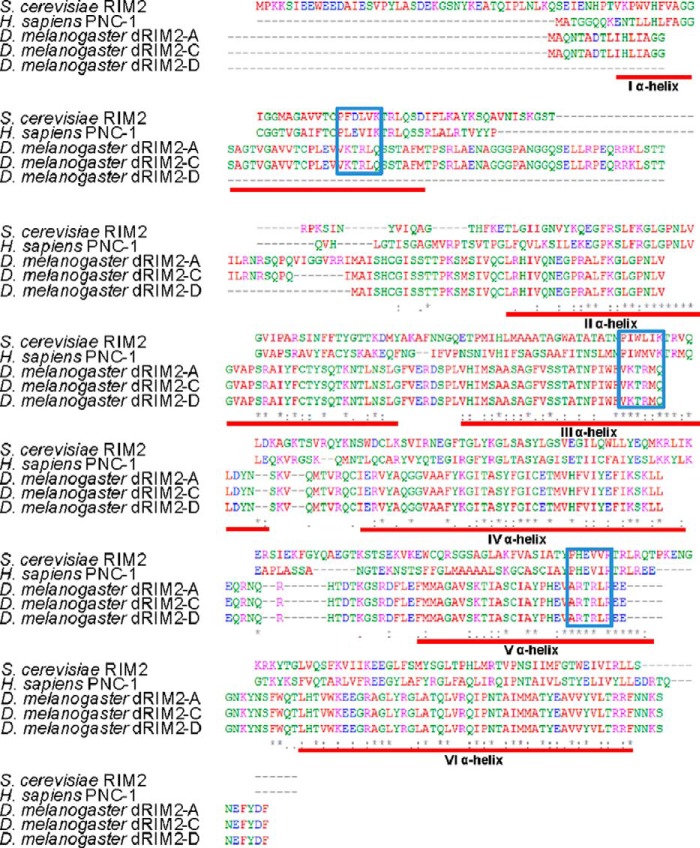
**Sequence alignment of RIM2 proteins from different species.** Sequences included in the alignment, performed with Multiple Sequence Alignment T-Coffee, are those of *S. cerevisiae*, *Homo sapiens* (SLC25A33), and *D. melanogaster* (dRIM2 isoforms A, C, and D). The alignment highlights identical residues (*) and similar ones (. and :), the positions of the six transmembrane α-helices (*red lines*), and the three signature motifs (*blue boxes*).

The Rim2p protein was previously characterized in *S. cerevisiae* as a mitochondrial pyrimidine nucleotide transporter (9). Yeast Rim2p is a member of the mitochondrial carrier protein family distinguished by some typical features as follows: the amino acid sequences include three repeats, each containing two putative trans-membrane sequences and the signature motif (P*X*(DE)*XX*(KR)). Therefore, the main structure fold is a six α-helical bundle (11). To evaluate the degree of conservation of the Rim2p across species, we performed an amino acid alignment of yeast, human, and fruit-fly Rim2p sequences and calculated the levels of homology (Fig. 1). The d*rim2* gene of *D. melanogaster* codes for three different transcripts, named A, C, and D. The corresponding protein isoforms showed comparable degrees of similarity to the yeast sequence (≈40%). Interestingly, all *Drosophila* isoforms are closer to human PNC-1 (52–54% identity) than to yeast Rim2p.

**FIGURE 2.**
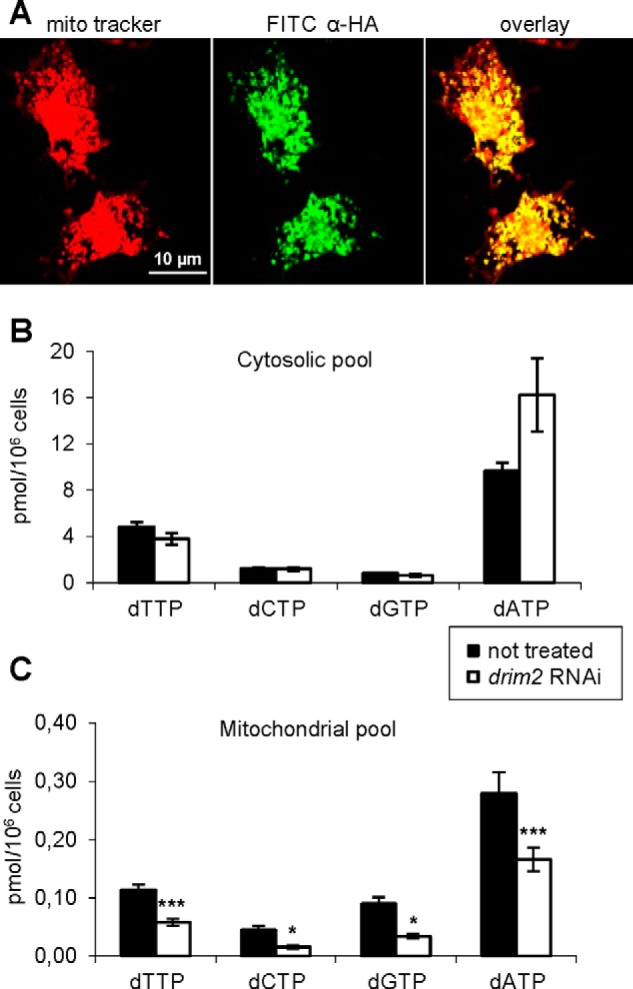
**Mitochondrial localization of dRIM2 and effects of d*rim2* silencing on the deoxynucleotides pool contents of S2R^+^.** *A, Drosophila* cells were transiently transfected with pACT-d*rim2* HA tagged, incubated with MitoTracker dye (*red*), and then immunolabeled with the anti-HA antibody (*green*). Overlay of images confirms the mitochondrial localization of dRIM2. Pyrimidine and purine dNTP pool sizes in cytosol (*B*) and mitochondria (*C*) of d*rim2* silenced S2R^+^ cells and controls. Pool sizes are expressed as picomoles of dNTP per million cells. Values are means ± S.D. from four experiments for control and five experiments for RNAi (Student's *t* test *, *p* < 0.05; ***, *p* < 0.005).

To characterize the subcellular localization of dRIM2, we transfected *Drosophila* S2R^+^ cells with a pACT vector expressing HA-tagged dRIM2 cDNA under the control of the *actin 5c* promoter. MitoTracker staining and immunodetection of HA-dRIM2 with an α-HA monoclonal antibody clearly indicated that dRIM2 localizes to mitochondria (Fig. 2*A*).

Next, we down-regulated the expression of endogenous d*rim2* in S2R^+^ cells and tested the effects on the size of the mitochondrial dNTP pools. In cells incubated for 72 h with dsRNA, real time RT-PCR analysis showed that mRNA was decreased by 80%. If dRIM2 is a carrier for pyrimidine nucleotides, its down-regulation should affect the dTTP and dCTP pools more than the purine deoxynucleotides dATP and dGTP. To test this prediction, we isolated the mitochondrial and cytosolic dNTP pools from control and silenced S2R^+^ cells by adapting a procedure previously devised for the quantification of mammalian mitochondrial dNTPs (19). No data are available on dNTP pool sizes in *Drosophila* cells, and we therefore wished to establish the relative abundance of the four dNTPs and the ratios between cytosolic and mitochondrial pools. In mammals, dTTP is generally the largest dNTP pool, and dGTP is the smallest, with dCTP and dATP occupying intermediate positions. Pool sizes in *Drosophila* are reported in Fig. 2, *B* and *C*. As in mammalian cells (22), mitochondrial pool sizes corresponded to about 3–10% those of the cytosolic pools. The dATP pool was the largest in both cytosol and mitochondria, followed by dTTP, dGTP, and dCTP in the cytosol and dTTP, dCTP, and dGTP in mitochondria. In both compartments, the dCTP pool was particularly small and comparable in size to the dGTP pool. Because the sizes of the dNTP pools are strongly influenced by the position of the cell in the cell cycle (36), we took care of comparing the concentrations of dNTPs in cultures of d*rim2*-silenced and control S2R^+^ cells with similar frequencies of S-phase cells. We observed no difference in the proportion of S-phase cells between silenced and control cultures, with values of about 20–25% depending on the experiment. Therefore, we feel confident that the differences in dNTP pools we measured in the two sets of cultures were not caused by differences in cell cycle distribution. However, although the cytosolic pools were virtually identical in control and silenced cultures (with the exception of the dATP pool that was higher in the latter), all mitochondrial dNTPs were significantly lower in the silenced cells, with levels ranging between 30% (dCTP) and 60% (dATP) of the controls. Thus, down-regulation of d*rim2* reduced the mitochondrial concentrations of both pyrimidine and purine dNTPs, suggesting that the protein is a general transporter for all four DNA precursors. We cannot extend this conclusion to RNA precursors, as we did not measure the mitochondrial ribonucleotide pools.

**FIGURE 3.**
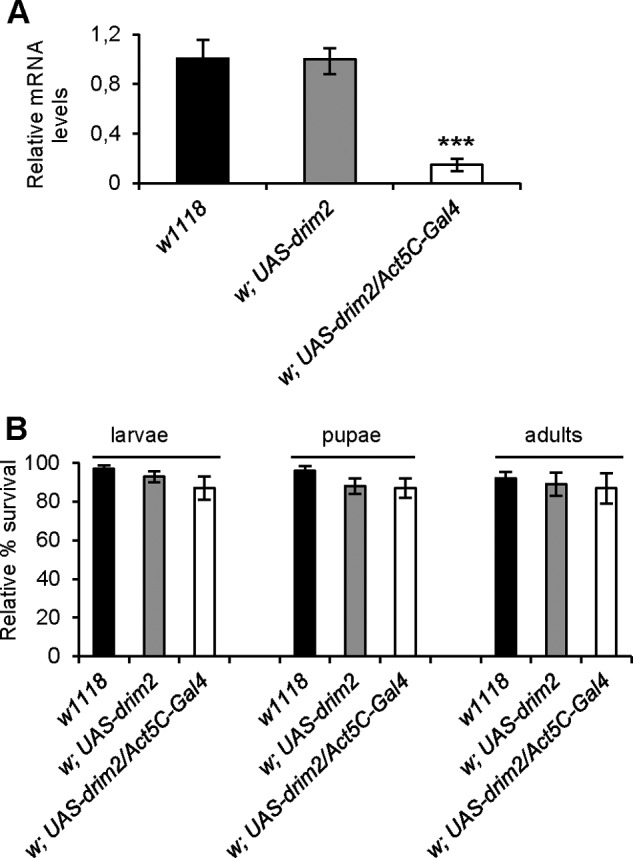
**Developmental effects of d*rim2* KD *in vivo*.** All experiments were carried out in *w*^1118^ (*closed column*) as well as in specific controls *w;UAS-*d*rim2* (*gray column*) and in KD *w;UAS-*d*rim2/Act5C-Gal4* (*open column*) third stage larvae. *A*, d*rim2* mRNA levels in each strain measured by qRT-PCR. *B*, relative percentage of egg to adult viability calculated at three developmental stages, *i.e.* third stage larvae, pupae, and adults. Data plotted are means ± S.D. (Student's *t* test ***, *p* < 0.005).

Next we attempted to knock down the d*rim2* gene *in vivo* by using GAL4/UAS-driven RNAi in living flies (18, 37). Despite high levels of silencing (about 80%) (Fig. 3*A*), d*rim2* KD individuals did reach the adult stage and lived longer than the controls (Fig. 3*B*). No effects on egg to adult viability were observed. These results suggest that even a low residual level of d*rim2* mRNA is sufficient to maintain the wild-type phenotype.

**FIGURE 4.**
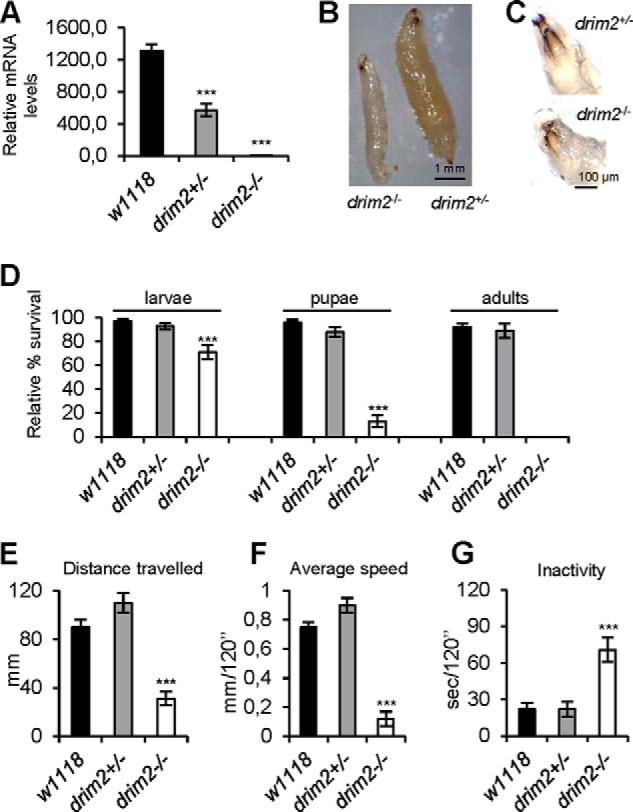
**Morphological, developmental, and behavioral effects of d*rim2* KO *in vivo*.** All experiments were carried out in parallel in *w*^1118^ (*closed column*), d*rim2*^+/−^ (*gray column*), and d*rim2*^−/−^ (*open column*) third stage larvae. *A*, d*rim2* mRNA levels, expressed as relative quantity of template in the sample, were determined by qRT-PCR. *B*, third stage KO larvae. Heterozygous d*rim2*^+/−^ larva (*right*) and homozygous d*rim2*^−/−^ larva (*left*). Notice the smaller size of the latter. *C*, both hetero- and homozygous KO larvae present mouth hooks, the distinctive characters of the third larval stage. *D*, relative percentage of egg to adult viability calculated at three developmental stages, *i.e.* third stage larvae, pupae, and adults. *E–G*, larval locomotor activity characterized by the three parameters, each calculated in a total recording time period of 120 s. *E,* total distance traveled (*i.e.* as millimeters covered); *F,* overall average speed (millimeters over 120 s); and *G,* inactivity (seconds over 120 s). Data plotted are means ± S.D. (Student's *t* test ***, *p* < 0.005).

Thus, we generated a *Drosophila* d*rim2* KO using the technique described previously (26) that exploits the specific recombination between FRT sites in the presence of flippase. Heterozygous KO flies (d*rim2*^+/−^) were balanced with a strain expressing GFP (w; L2, Pin1, *CyO-*GFP), allowing discrimination between homozygous GFP-negative d*rim2*^−/−^ and heterozygous GFP-positive d*rim2*^+/−^ larvae. Real time-PCR showed that d*rim2*^+/−^ larvae had about 50% d*rim2* mRNA levels compared with a wild-type control (*w*^1118^), whereas d*rim2*^−/−^ flies were null, as expected (Fig. 4*A*). The d*rim2*^−/−^ third instar larvae were visibly smaller than their heterozygous counterparts (Fig. 4*B*). Nevertheless, KO larvae did present mouth hooks, the distinctive character of the third larval stage indicating that their smaller size was not due to a developmental delay (Fig. 4*C*). The KO heterozygous d*rim2*^+/−^ larvae developed into normal adults with no developmental defects. On the contrary, none of d*rim2*^−/−^ larvae reached adulthood. Although d*rim2*^−/−^ individuals survived through larval development, most of them died at the third larval instar, and the survivors failed to progress beyond the pupal stage (Fig. 4*D*). To further characterize the phenotype, we measured locomotor activities (total distance traveled, overall average speed, and inactivity) with the Any Maze software. Wild-type and d*rim2*^+/−^ larvae behaved similarly, whereas d*rim2*^−/−^ showed marked locomotor defects (Fig. 4, *E–G*).

**FIGURE 5.**
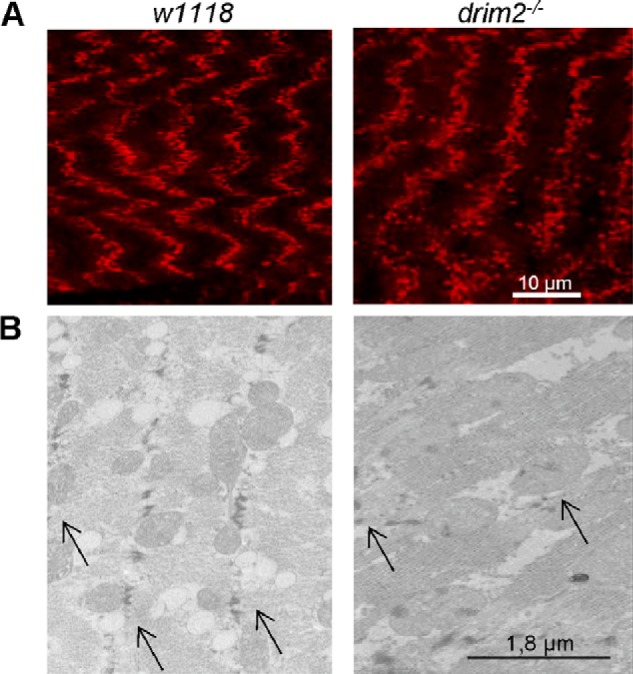
**Mitochondria disposition around z-lines.** Confocal images (*A*) and electron microscopy (*B*) of body wall preparations of *w*^1118^ and d*rim2*^−/−^ larvae. *Arrows* indicate the z-lines.

**FIGURE 6.**
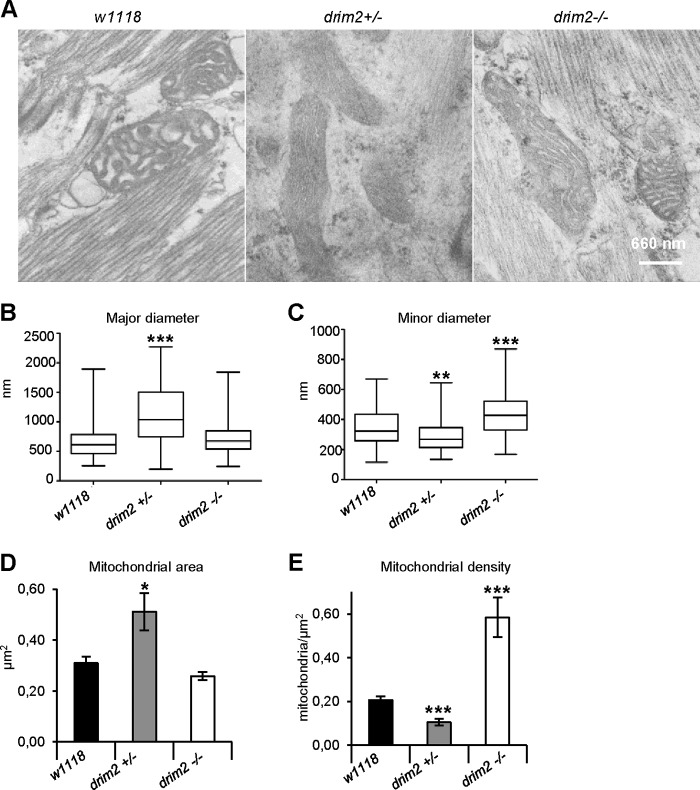
**Electron microscopic analysis on third stage larval body wall sections.** Characterization was carried out on *w*^1118^, d*rim2*^+/−^, and d*rim2*^−/−^ larvae. *A*, cross-sectional ultrastructure of larval muscles, illustrating the distribution and morphology of mitochondria. *B* and *C,* morphometric analyses of mitochondrial dimensions in terms of minor (*B*) and major diameter (*C*), both expressed in nanometers and presented as box plots. *D*, mean ± S.E. of the total area occupied by mitochondria (expressed in square microns) over the tissue profile area measured (*w*^1118^, 447 μm^2^; d*rim2*^+/−^, 231 μm^2^; d*rim2*^−/−^, 256 μm^2^). *E*, mitochondria density ± S.E. was plotted as the number of mitochondria per square micron of the tissue profile area (*w*^1118^, 447 μm^2^; d*rim2*^+/−^, 231 μm^2^; d*rim2*^−/−^, 256 μm^2^) (Student's *t* test *, *p* < 0.05; **, *p* < 0.01; ***, *p* < 0.005).

Confocal images of body wall preparations stained with MitoTracker Red showed a normal mitochondrial pattern along the z-lines in *w*^1118^; however, the d*rim2*^−/−^ individuals showed spatially disorganized mitochondria that failed to line up along the z-lines (Fig. 5*A*). This abnormal pattern was confirmed by transmission electron microscopy (EM) (Fig. 5*B*). EM carried out in d*rim2*^+/−^ and d*rim2*^−/−^ larvae (*1st panel,* Fig. 6*A*) revealed alterations of mitochondrial number and shape compared with *w*^1118^ (Fig. 6*A*). The d*rim2*^+/−^ mitochondria appeared more elongated and considerably bigger than those of *w*^1118^ larvae (Fig. 6*A*). Morphometric analysis indicated that, on average, the major mitochondrial diameter in d*rim2*^+/−^ larvae was significantly increased, and the minor diameter was reduced relative to *w*^1118^ mitochondria (Fig. 6, *B* and *C*). In d*rim2*^−/−^ larvae, mitochondria had a rounder shape (Fig. 6*A*); the averaged major diameter was unchanged relative to the wild type, whereas the minor diameter was longer than that of mitochondria of both *w*^1118^ and d*rim2*^+/−^ (Fig. 6, *B* and *C*). Accordingly, estimates of the mitochondrial area indicated that d*rim2*^+/−^ organelles were larger than those from *w*^1118^ and d*rim2*^−/−^ flies (Fig. 6*D*). Furthermore, mitochondrial density, *i.e.* the number of mitochondria per surface unit, was reduced in d*rim2*^+/−^ individuals compared with wild-type controls and significantly higher in d*rim2*^−/−^ larvae (Fig. 6*E*).

**FIGURE 7.**
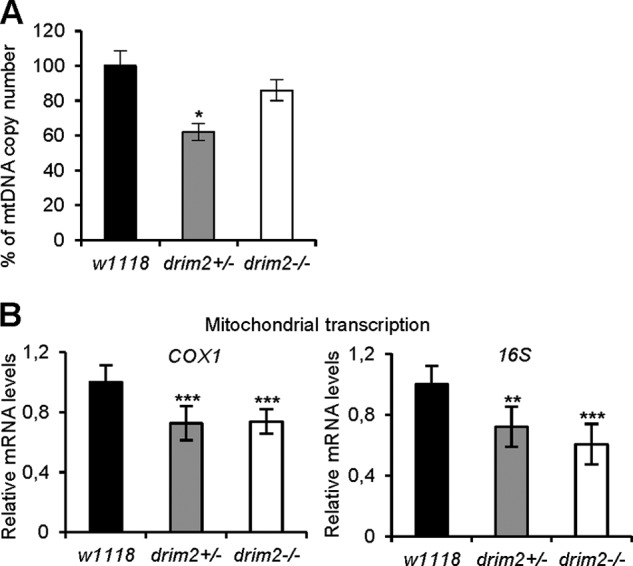
**Mitochondrial DNA content and analysis of mitochondrial transcription in larvae.** All experiments were carried out on *w*^1118^, d*rim2*^+/−^, and d*rim2*^−/−^ larvae. *A*, mtDNA content was measured by quantitative real time-PCR. Data are ratios of mtDNA to genomic DNA relative to the control ratio in *w*^1118^ larvae. Values are expressed as a mean of three independent experiments, and *error bars* represent the S.D. of the mtDNA/nDNA ratio among the replicates. *B*, analysis of mitochondrial transcription. Transcripts of the mitochondrial genes *COX1* and *16S* were measured by real time-PCR and related to the level of transcripts of the nuclear gene *Rpl32*. For each condition, data are presented as the mean of relative quantity of template in the sample ± S.D. from three individual experiments (Student's *t* test *, *p* < 0.05; **, *p* < 0.01; ***, *p* < 0.005).

As *Rim2* deletion in yeast and PNC1 down-regulation in mammalian cells decreases mtDNA content (13, 14), we measured the mtDNA copy number in KO larvae. Unexpectedly, although mtDNA was almost 40% depleted in d*rim2*^+/−^ larvae, d*rim2*^−/−^ individuals had levels of mtDNA close to wild type (Fig. 7*A*), possibly a consequence of the higher mitochondrial density (Fig. 6*E*) (38, 39).

**FIGURE 8.**
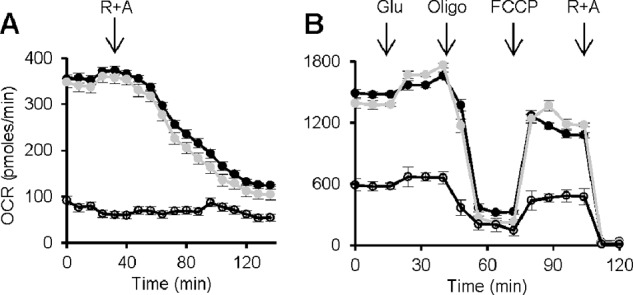
**Oxygen consumption rates in larvae and in S2R^+^ cells.** *A*, oxygen consumption was measured in *w*^1118^ (*black vehicles*), d*rim2*^+/−^ (*gray vehicles*), and d*rim2*^−/−^ (*empty vehicles*) third stage larvae. The addition of 5 μm rotenone (*R*) plus 5 μm antimycin A (*A*) is indicated by the *arrow. B*, respiratory profile of control and d*rim2*-silenced S2R^+^ cells. We analyzed untreated cells (*black vehicles*), cells silenced for 48 h with dsRNA (*gray vehicles*), and cells silenced for 96 h (*empty vehicles*). Ten mm glucose (*Glu*), 1 μm FCCP, and 5 μm rotenone plus 5 μm antimycin A were added at the times marked by *arrows*.

A reduced mtDNA copy number might lead to reduced expression of the mtDNA-encoded subunits of the respiratory chain. We measured the levels of mitochondrial transcripts for *Cyclooxygenase-1* (*COX1*) and RNA (*16S*) relative to housekeeping gene *Rpl32* by real time-PCR (Fig. 7*B*) (40). Mitochondrial transcription was lower in both d*rim2*^−/−^ and d*rim2*^+/−^ larvae. To establish whether defects in d*rim2* function also affect mitochondrial respiration, we measured oxygen consumption rates of muscle body wall preparations of *Drosophila* larvae with the Seahorse technology (41). Controls and d*rim2*^+/−^ larvae maintained a steady respiratory rate that was inhibited by rotenone and antimycin A, demonstrating its mitochondrial origin. On the contrary d*rim2*^−/−^ larvae showed severe impairment of oxygen utilization that was insensitive to respiratory inhibitors (Fig. 8*A*). The same measurements were performed also in S2R^+^ cells silenced for d*rim2*. The rate of oxygen consumption was significantly decreased in cells silenced for 96 h, and the cells responded less than the controls to uncoupler (FCCP) and to oligomycin or rotenone plus antimycin A (Fig. 8*B*). No significant differences of respiratory profile were detected between cells undergoing a mock interfering treatment for almost 48 h and the controls. The basal respiration of the two cultures was similar, both being of mitochondrial origin because they were stimulated by FCCP and inhibited by oligomycin or rotenone plus antimycin A (Fig. 8*B*).

**FIGURE 9.**
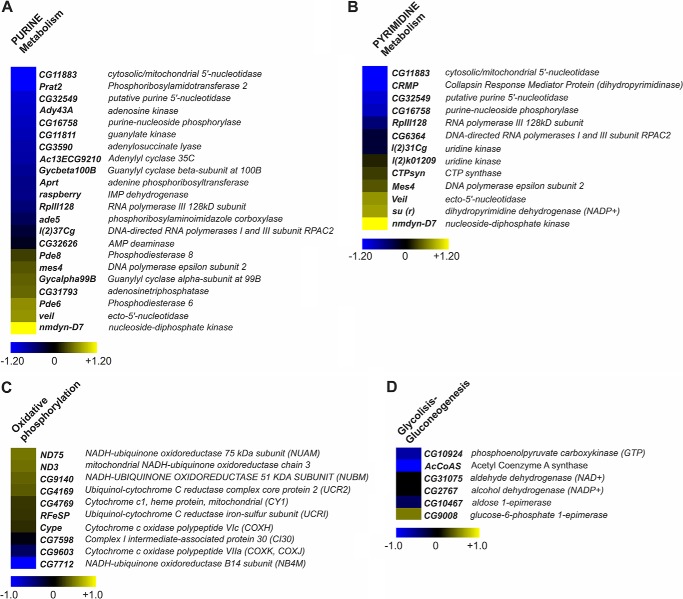
**Altered gene pathways in d*rim2* KO *Drosophila*.** Heat map representing a selection of deregulated transcripts, provided by DAVID tool, in d*rim2*^−/−^ *versus* d*rim2*^+/−^ involved in purine (22 transcripts) and pyrimidine (13 transcripts) metabolism (*A* and *B,* respectively), as well in oxidative phosphorylation (10 transcripts) and glycolysis/gluconeogenesis (six transcripts) (*C* and *D,* respectively). A color-coded scale for the normalized expression values is used as follows: *yellow* and *blue* represent high and low expression levels in d*rim2*^−/−^ with respect to d*rim2*^+/−^, respectively. The expression level of each transcript was calculated as the log2 (d*rim2*^−/−^*/* d*rim2*^+/−^), and the complete list of differentially expressed genes identified by Significance Analysis of Microarray algorithm is provided in the supplemental Table S1.

To define the gene expression pattern specifically associated with d*rim2* KO, we performed protein-coding microarray analyses (Fig. 9) (*Drosophila* 1.0 custom platform, Agilent Technologies) on high quality RNA from d*rim2*^−/−^ and d*rim2*^+/−^. Using Significance Analysis of Microarray two class analysis, we identified 2,964 differentially expressed genes (false discovery rate = 5.10%) of which 1,120 were up-regulated (38%) and 1,844 were down-regulated (62%) in d*rim2*^−/−^ *versus* d*rim2*^+/−^ samples (supplemental Table S1). A functional annotation web tool (DAVID) was used to identify functional categories occurring in the d*rim2*^−/−^ expression signature more frequently than expected by chance. The Group Enrichment Score was used to rank biological significance of deregulated genes. We observed that Gene Ontology functional categories over-represented in the up-regulated component of the expression signature included oxidative phosphorylation and glycolysis/gluconeogenesis; in contrast, the down-regulated components showed an over-representation of Gene Ontology categories such as purine and pyrimidine metabolism (Fig. 9).

## DISCUSSION

Mitochondria contain multiple copies of a small circular DNA coding for essential polypeptide components of the respiratory chain and F-ATP synthase complexes embedded in the inner mitochondrial membrane. Replication and transcription of mtDNA occur during the whole life of the cell, even after it has reached terminal differentiation. Both processes are particularly active during cell proliferation and early development, when mitochondrial biogenesis is induced (42) and thus the request for dNTPs and rNTPs is particularly high. In yeast and mammals, the cytoplasm is the main site for nucleotide production, but some synthesis also occurs in mitochondria and in the nucleus (4, 5). Because of the impermeability of the mitochondrial inner membrane to nucleotides, these are taken up into mitochondria by a partly unknown repertoire of membrane carriers. The nucleotide transporter we have studied here is the *Drosophila* ortholog of yeast Rim2p and human PNC1, two homologous pyrimidine nucleotide transporters (9, 11, 12).

In yeast, the genetic inactivation of RIM2 causes loss of mtDNA and a petite phenotype (13). In cultured human cells, down-regulation of PNC1 by siRNA can lead to decreased mtDNA copy number (14). Thus, in both yeast and humans the dependence of mitochondria on nucleotides made in the cytosol has been demonstrated.

The study of nucleotide, and especially deoxynucleotide, metabolism has been relatively neglected in *Drosophila*. Most attention has been dedicated to a distinctive multisubstrate deoxynucleoside kinase responsible for the salvage of all deoxynucleosides (43), as opposed to the four separate deoxynucleoside kinases existing in mammals (6). Given the presence in the genome of the key enzymes for dNTP and rNTP *de novo* synthesis, we assumed that the general picture defined in mammalian cells also applies to *Drosophila* and that also in this species mitochondria obtain nucleic acid precursors from extra-mitochondrial sources. Thus, after confirming that dRIM2 is localized to mitochondria (Fig. 2*A*), we hypothesized that its deletion may impact the mtDNA content and impair mitochondrial transcription, with negative consequences for oxidative phosphorylation and energy-dependent processes.

We studied the effects of dRIM2 ablation on the dNTP pools required for the maintenance of mtDNA. No information was available on the composition of dNTP pools of *Drosophila* cells, and we first analyzed the total pools of control S2R^+^ cells. They contained relatively more dTTP and dATP than dCTP and dGTP, a pool composition different from that commonly observed in mammalian cells where dTTP and dCTP frequently are the most abundant dNTPs. When we separated mitochondrial and cytosolic pools we found that, in *Drosophila* as in human cells, the dNTP pools of mitochondria amount to no more than 10% of the total dNTPs. In d*rim2*-silenced cells, both mitochondrial pyrimidine and purine dNTPs pools were reduced. Although these data only give a static picture of the mitochondrial pools and the actual nucleotide transport was not directly measured, the general decrease of all four dNTPs suggests that dRIM2 acts as a general deoxynucleotide transporter. Cytosolic pools were unaffected, underscoring a specific function of the carrier in the import of nucleotides from the site of their synthesis in the cytoplasm into the mitochondrial matrix where they are consumed for mtDNA synthesis. The possible lack of intramitochondrial dNTP synthesis in *Drosophila* suggested by the genomic data is supported by the appearance of cell toxicity when silencing was prolonged beyond 3 days. Although the treatment did not completely remove d*rim2* mRNA, the down-regulation was sufficient to impair cell viability *in vitro*. This was not the case when we down-regulated the protein *in vivo* using the GAL4/UAS system to ubiquitously activate d*rim2*-dsRNAi. A 30% residual level of gene activity was sufficient for the flies to develop normally into adults (Fig. 3).

A very different picture appeared after *in vivo* KO of d*rim2.* Homozygous d*rim2*^−/−^ larvae exhibited a lethal phenotype and died during the third larval stage without reaching adulthood. The homozygous KO produced an overall impairment of larval development associated with reduced dimensions compared with wild-type larvae (Fig. 4, *B–D*). It is known that most *Drosophila* mutants for genes involved in mitochondrial and nucleotide metabolism either do not undergo metamorphosis, arresting their development at larval stage or manifest developmental defects. This phenomenon is probably related to the particularly high energetic burden of metamorphosis. Larval lethality is frequently associated with neuromuscular and behavioral defects, functions that in *Drosophila* are particularly sensitive to OXPHOS-dependent energy drop (44–46). Here, d*rim2*^−/−^ larvae showed severe defects in their locomotor activity (Fig. 4, *E–G*).

From the morphological point of view, mitochondria of both d*rim2*^−/−^ KO and heterozygous d*rim2*^+/−^ larvae displayed evident anomalies (Fig. 6). EM analysis in d*rim2*^−/−^ larvae revealed a higher mitochondrial density (Fig. 6*E*), possibly a physiological response compensating for the progressive loss of mitochondrial function. However, expression profiling did not demonstrate activation of mitochondrial biogenesis. Genes such as *PGC-1*α, *NRF-1*, *NRF-2,* and *TFAM* were not up-regulated in d*rim2*^−/−^ larvae. An alternative explanation for the increased number of mitochondria may be the reduced mitochondrial turnover rather than increased biogenesis.

Interestingly, among the genes involved in nucleotide metabolism that are differentially expressed in homozygous KO larvae, only nucleoside diphosphate kinase was clearly up-regulated, and all others were down-regulated. Because nucleoside diphosphate kinase catalyzes the final step in the synthesis of dNTPs and rNTPs, this result suggests a homeostatic response to reduced availability of nucleoside triphosphates in mitochondria. The down-regulated genes encode both for synthetic and catabolic enzymes and participate in the regulation of different pool components in combination with other enzymes. Changes detected in individual members of a complex enzyme network are difficult to interpret and may reflect general unspecific stress rather than a concerted metabolic adjustment related to the lack of mitochondrial nucleotides.

Despite the severity of the KO phenotype, direct quantitation of mtDNA in the d*rim2*^−/−^ larvae did not show the expected depletion (Fig. 7*A*) possibly due to their observed higher mitochondrial density (Fig. 6*E*). Moreover, d*rim2*^−/−^ larvae show a decrease of mitochondrial transcripts (Fig. 7*B*). These results indicate that d*rim2* and *PNC1* are necessary for mtDNA transcription and replication in flies and humans, respectively.

Mitochondrial disorders are characterized by impaired oxygen consumption, and homozygous KO larvae and d*rim2-*silenced cells are no exception (Fig. 8), as expected on the basis of the reduced mtDNA copy number and transcription.

Yeast *Rim2* was identified in screens for suppressors of high iron toxicity in strains deleted for the two yeast mitochondrial iron transporters *Mrs3* and *Mrs4* or for the vacuolar iron transporter *CCC1* (47, 48). Thus, the protein was proposed to play a dual role as pyrimidine nucleotide and iron transporter. However, recent data show that in wild-type yeast deletion of RIM2 alone is irrelevant for mitochondrial iron supply (49). The *Drosophila* genome contains *mfrn,* the gene for mitoferrin that is the homolog of the yeast iron carriers Mrs3 and -4. Therefore, we assume that the effects of d*rim2* KO detected here depend primarily on the lack of nucleotide transport into mitochondria.

Our findings strongly suggest that the *D. melanogaster CG18317* (d*rim2*) gene is essential to maintain mitochondrial function by providing deoxynucleotides for mtDNA transactions. dRIM2 is the first (deoxy)nucleotide carrier characterized in *Drosophila,* and our KO larvae are the first animal model of RIM2 deficiency. The data presented here may offer a key to understand the functional role of RIM2 in a multicellular animal and further support a general function in deoxynucleotide transport in mitochondria.

## Footnotes

3 The abbreviations used are:

mt: mitochondrial
qRT-PCR: quantitative PCR
FCCP: carbonyl cyanide *p*-trifluoromethoxyphenylhydrazone
F: forward
R: reverse.
